# Vitiligo International Task force for an Agreed List of core data (VITAL): study protocol of a vitiligo core outcome set (COS) and contextual factors for clinical trials, registries, and clinical practice

**DOI:** 10.1186/s13063-022-06497-1

**Published:** 2022-07-23

**Authors:** Nanja van Geel, Iltefat H. Hamzavi, Amit G. Pandya, Albert Wolkerstorfer, Julien Seneschal, Amit Garg, Phyllis Spuls, Caroline B. Terwee, Sue Mallett, Reinhart Speeckaert, Jean Marie Meurant, Viktoria Eleftheriadou, Khaled Ezzedine

**Affiliations:** 1 Department of Dermatology, Ghent University Hospital, Ghent, Belgium; 2 Department of Dermatology, Henry Ford Hospital, Global Vitiligo Foundation, Detroit, MI USA; 3 Department of Dermatology, The University of Texas Southwestern Medical Center, Dallas, TX USA; 4 Department of Dermatology, Amsterdam Public Health/Infection and Immunology, Location AMC, A0-227, University of Amsterdam, Amsterdam, The Netherlands; 5 Department of Dermatology, INSERM U 1035, University of Bordeaux, Bordeaux University Hospitals, Bordeaux, France; 6 Department of Dermatology, Donald and Barbara Zucker School of Medicine at Hofstra/Northwell, New Hyde Park, NY USA; 7 Department of Epidemiology and Data Science, Amsterdam University Medical Centers, Vrije Universiteit Amsterdam, Amsterdam Public Health Research Institute, Amsterdam, The Netherlands; 8 UCL Centre for Medical Imaging, University College London, London, UK; 9 Vitiligo International Patient Organizations, 11 rue de Clichy, 75009 Paris, France; 10 New Cross Hospital, The Royal Wolverhampton NHS Trust, Wolverhampton, UK; 11 Department of Dermatology, University Hospital Henri Mondor, EpiDermE EA 7379, Université Paris-Est Créteil Val de Marne, Créteil, France

**Keywords:** Core dataset, Vitiligo, e-Delphi, Domains, Measurement instruments, Core outcome set

## Abstract

**Background:**

There is a lack of consensus related to the collection of standardized data for individuals with vitiligo enrolled in clinical trials and registries as well as those seen in clinical practice which causes difficulty in accurately interpreting, comparing, and pooling of data.

Several years ago, efforts to initiate work on developing core outcome sets were performed and a consensus was reached in 2015 on the first core domain set for vitiligo clinical trials.

**Methods/design:**

This project aims to further develop a core outcome set for vitiligo clinical trials as well as create internationally agreed-upon core outcome sets for registries and clinical practice. These core outcome sets will include a core domain set and a core measurement instruments set and will be supplemented by contextual factors, including baseline and treatment-related characteristics. In a preparatory exercise, the 2015 core domain set will be re-evaluated and will serve as the basis for the list of outcome domains used to initiate the consensus process. This project will consist of two parts. Part 1 will focus on the selection of a core domain set, or “what to measure” and contextual factors, for each setting based on electronic surveys (e-Delphi technique) and a conclusive consensus meeting by a large group of international stakeholders. Part 2 will include selection of core measurement instruments, or “how to measure,” and measurement details (e.g., scale and timing) for the core domain sets and contextual factors agreed upon in part 1. Part 2 will be based on consensus meetings with stakeholders involved in part 1 and will be guided by C3 (CHORD-COUSIN Collaboration), Harmonising Outcome Measures for Eczema (HOME), COnsensus-based Standards for the selection of health Measurement INstruments (COSMIN), and Outcome Measures in Rheumatology (OMERACT) recommendations including information on measurement properties of available instruments (systematic review and expert/patient opinion). At the end of part 2, all stakeholders involved will be invited to participate in a final meeting in which the ultimate core data sets (core outcome sets and contextual factors) will be presented and the dissemination plan and implementation goals will be defined.

**Discussion:**

This project will harmonize data collection between clinical trials, registries, and clinical practices, facilitating new insights in vitiligo.

**Trial registration:**

This study is registered in the Core Outcome Measures for Effectiveness Trials (COMET) database and on the C3 (CHORD-COUSIN Collaboration) website.

## Background

Vitiligo affects approximately 1% of the world population and is being actively investigated for novel treatments. However, collecting all relevant data in a standardized, reliable, and feasible manner is still a challenge for this condition. Standardized outcomes are relevant in the context of vitiligo epidemiology, management, estimation of prognosis, understanding of disease progression, development of therapeutic options, distribution of resources, and pharmaco-economic evaluations. In 2012, two systematic reviews on vitiligo outcome measures used in clinical trials identified 25 different domains. These domains were measured and reported using a variety of different instruments and scales (e.g., repigmentation alone was reported by 48 different scales) and information on the measurement properties of instruments was limited [1, 2]. Moreover, there continues to be a lack of consensus on standardized data collection to monitor vitiligo in trials and clinical practice [3]. This makes it difficult to accurately interpret, compare and pool the data across trials and hampers the development of clinical guidelines. As such, this problem has direct implications in the management of vitiligo patients.

The Vitiligo European Task Force (VETF) of the European Academy of Dermatology and Venereology (EADV), the Vitiligo Global Issues Consensus Conferences (VGICC) group, and the Initiative for outcome measures in vitiligo (INFO) in cooperation with the Core Outcome Measures in Effectiveness Trials (COMET)-initiative reached international consensus in a multi-perspective web-based Delphi (e-Delphi) on the core domains/domain items for clinical trials in vitiligo, or “what to measure” [4]. Three outcome domains/domain items were deemed “essential” to be measured in every vitiligo trial relevant to all vitiligo treatments: repigmentation, maintenance of repigmentation, and side effects. Four other outcomes were “recommended” to be measured where relevant and applicable in vitiligo trials: cosmetic acceptability of the results, cessation of spreading, tolerability or burden of treatment, and quality of life.

Following the core domain set agreement for trials, three large patient workshops were conducted in collaboration with the Global Vitiligo Foundation/Global Vitiligo Foundation Support Community (GVF/GVFSC) in the USA with an aim to define successful repigmentation of a target lesion from the patient’s point of view and propose how and when repigmentation in target lesions should be evaluated in clinical trials in vitiligo [5].

Guidance from the CS-COUSIN and the Vitiligo Global Issues Consensus Group was followed and the recommendations were to use the percentage of repigmentation in quartiles (0–25%, 26–50%, 51–79%, 80–100%) and the Vitiligo Noticeability Scale as outcome measurement, based on the best available evidence at that time.

This project aims to further develop international consensus on a core outcome set in vitiligo clinical trials for all vitiligo treatments, applicable to full-body/regional evaluation and in a second step for target lesions, as well as create internationally agreed-upon core outcome sets for registries and clinical practice. These core outcome sets will include a core domain set (“what to measure”) and a core measurement instruments set (“how to measure”) and will be supplemented with contextual factors, including baseline and treatment-related characteristics, where relevant.

In addition to measurement instruments, the timing (“when to measure”), scales, and instructions for standardized reporting [using the reporting guidelines of the EQUATOR network (e.g., CONSORT checklist as a guide)] will be defined. The ultimate, long-term goal of this project is to enable all researchers and clinicians to collect data in a standardized manner.

## Methods

### Summary, parts 1 and 2

**Fig. 1:**
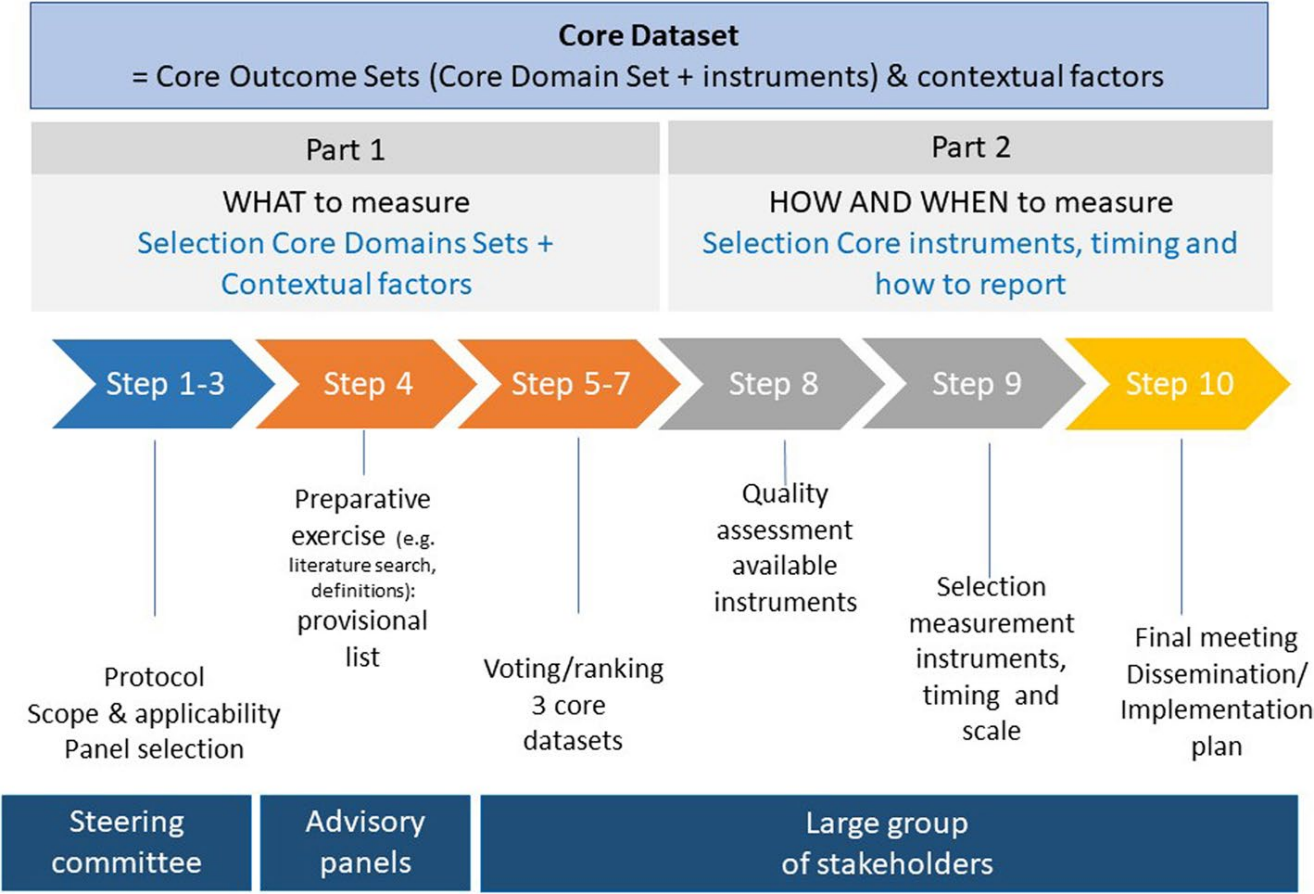
Overview including 10 steps of the project

This project will consist of two parts and includes 10 different steps (Fig. 1 and Table 1). Part 1 will focus on “what to measure” and will include a preparative stage [including evaluation of previously (2015) defined core domains/domain items for trials and formulation of definitions], electronic surveys (e-Delphi technique), and a conclusive consensus meeting to select a Core Domain Set (core domains/domain items) and contextual factors (baseline and treatment-related characteristics) for clinical trials, registries, and clinical practice separately. A large group (±100) of international stakeholders will be involved, including vitiligo experts, vitiligo researchers, patients, patient representatives, and other stakeholders, such as industry and regulatory representatives and journal editors. Three separate smaller advisory panels (±15–20 stakeholders per panel for each setting including patients) will be created to support various aspects of this project. In addition, patient focus groups will be assembled from patients involved in the advisory panels and/or patients recruited by vitiligo patient organizations. Part 2 will focus on “how to measure” and will include the selection of the core instruments, measurement details (e.g., scale and timing), and instructions for standardized reporting for the core domain sets and contextual factors (baseline and treatment-related characteristics) agreed upon in part 1. This will be based on consensus meetings using structured group discussions with preferably all stakeholders involved in part 1 [6–8].

**Table 1:** Ten steps of the project including intermediate goals (in bold) and the team involved

	Step	Content and intermediate goals (in bold) of each step	Who?
Part 1	1	Discussion with and **approval of the protocol** by steering committee and methodological collaborators [e.g. COSMIN, C3 (CHORD/COUSIN Collaboration)]	SC, M, ST
	2	Definition of **scope and applicability** for each setting	SC
	3	**Information, selection, and invitation of participant** [including advisory panels/patient (focus) groups]: e.g., health care professionals/researchers, patients, industry representatives and subsequent comprehensive explanation of the protocol to participants	SC, M
	4	**Development provisional lists** for outcome domains/domain items and contextual factors by steering committee and advisory panel based on preparative exercise: revision previous (2015) core domains for trials; definitions (patient focus groups/patient surveys), literature search + items used in existing clinical practice/registries + expert/patient opinions.	SC, PFG, AP
	5	Electronic surveys according to e-Delphi technique to score the importance of outcome domain items and contextual factors (maximum 3 rounds) by all participants.	APT
	6	A **conclusive meeting** will be organized to solve remaining disagreements or items with “no consensus.”	APT
	7	Process (steps 4–6) will be repeated for each setting to **construct 3 core domain sets** (clinical trials; registries; clinical practice)	APT
Part 2	8	Quality assessment (evaluation of measurement properties) of available instruments for vitiligo (PROMs, ClinROM, and imaging techniques) by COSMIN checklist (updated systematic review).	CG
	9	**Selection of measurement instruments and measurement details** (e.g., scale and timing) during consensus meetings guided by HOME and COSMIN recommendations for each setting.	APT
	10	A final meeting will be organized to present an overview of the composed 3 dataset and to define the dissemination plan and implementation goals.	APT

*SC*, steering committee; *M*, methodologist; *ST*: statistician; *AP*, advisory panels; *PFG*, patient (focus) groups; *APT*, all participants; *CG* core team Ghent; *PROMs*, patient-reported outcome measures; *ClinROM*, clinician-reported outcome measures

### Methodological techniques

Methodological overview of the different steps included in the project will be provided by members of the steering committee and methodologists. The methodology for the selection of the Core Outcome Sets within our Core Dataset will be guided by the Harmonising Outcome Measures for Eczema (HOME)-roadmap and the Core Outcome Measures in Effectiveness Trials (COMET) handbook. In addition, collaboration with C3 [Consortium for Harmonizing Outcomes Research in Dermatology (CHORD) and Cochrane Skin-Core Outcome Set Initiative (CS-COUSIN) - collaboration], COnsensus-based Standards for the selection of health Measurement INstruments (COSMIN) and COMET registration will further assure a high methodological quality of the project [9–14].

For the consensus process in PART 1 (“what to measure”), the e-Delphi method will be used, which is based on the ability to receive opinions from a large group of stakeholders in a structured fashion. This method will also support the uptake of the final product by a large group of stakeholders. Participants will be asked to follow this procedure for a maximum of 3 rounds.

The consensus process in part 2 (“how” to measure) will be based on a structured group discussion during meetings to reach a consensus [e.g., Nominal Group Technique (NGT)] as it will enable the collection and selection of a large amount of data in a defined period of time and facilitate problem-solving of remaining issues with a smaller group [6–8]. Based on previous experiences, the steering group considered a structured group discussion most suitable for the selection of instruments, scales, and timing.

### Methodology, step by step

#### Part 1

##### Step 1: Construction protocol

Based on virtual conference calls (January 2020–January 2022) the protocol was reviewed by the steering committee and methodologists. Modifications were included until an agreement was reached.

##### Step 2: Scope and applicability

In addition, the scope and applicability of the core datasets were defined by the steering committee [e.g., target population (condition and intervention), setting (e.g., clinical trials, registries, and clinical practice), and geographical/regional scope]). The target population for clinical trials is vitiligo (non-segmental), adults and children, all treatments; for registries: vitiligo (non-segmental), adults and children, all treatments, and no treatment; for clinical practice: vitiligo (non-segmental) and segmental vitiligo, adults and children, all treatments, and no treatment. For all settings, the geographical scope is “global.” Based on the defined scope and applicability, all participants will be instructed on the intended use of the core datasets during the different steps of the study.

##### Step 3: Selection of advisory panel members, general study participants, and patients/patient focus groups

For the selection of advisory panel members (small group of stakeholders) and general study participants (a large group of stakeholders) the steering group will invite vitiligo experts/researchers from dermatologic organizations [e.g., Vitiligo Task Force (VTF), Global Vitiligo Foundation (GVF), VGICC, and International League of *Dermatological* Societies (ILDS)], as well as patients from patient organizations and industry/regulatory representatives. The total number of general participants is unlimited, while each advisory panel will include a maximum of ±15–20 members: ±7–9 vitiligo experts/researchers (including a minimum presence of 3 steering committee members), ±4 patients*/*patient representatives (and ± 4 backup patients), and 1 other stakeholder (methodologist, journal editor, or industry representatives) or neutral observer. Selection of the general study participants and advisory panel members will be performed by the steering group keeping in mind the scope and setting (trials, registries, clinical practice) of this project. Criteria for selection are academic or clinical interest, expertise in vitiligo, and leading or nominated members of societies. Advisory panel members may participate in several panels to ensure consistency of the process. The selected advisory panel members will be invited by email including a description of the expected tasks [e.g., (1) provide data, (2) assist in the preparation of the surveys and the meetings (including the construction of the provisional list for e-Delphi), (3) support proofreading of documents]. Selection of patients/patients’ representatives for the advisory panels and patient focus groups/patient groups will be based on suggestions from patient advocacy organizations, such as the Vitiligo International Patient Organizations Conference (VIPOC), the Vitiligo Society UK, and Global Vitiligo Foundation Support Community (GVFSC). All participants of the study will be asked for their consent to take part in the study. They will be selected from different countries/continents (Europe, Africa, North America, South America, Asia, Australia).

Start-up meetings will be organized to explain the project in more detail. This will include meetings devoted to the advisory panels as well as a general introduction meeting for all general participants/patients. Specific attention will be paid to clarify the exact aim, different steps, and scope for each setting.

##### Step 4: Development of a provisional list of items to include in the first e-Delphi round

The aim of this step is to construct a provisional list of outcome domains/domain items as well as their definitions to start the voting rounds (e-Delphi). This provisional list of items may differ according to the setting (clinical trials, registries, clinical practice). Possible groups for outcome domains are (a) physician-reported outcomes (including vitiligo status variables and treatment response), (b) patient-reported outcomes (e.g., aspects of quality of life), and (c) objectively-measured outcomes (e.g., digital image analysis). This list will be composed by the steering committee and advisory panel members and based on a preparative exercise. This exercise will start with the 2015-defined outcome domains/domain items for trials and includes a check with patients, healthcare professionals (steering committee, advisory panel members), to establish if the previous core domain set is still up to date, needs modification, and anything vital is missing (content validity) for each setting separately. These previously defined (2015) core domains/domain items can subsequently be “re-categorized” (e.g., from “recommended” to “essential”) and renamed. Moreover, additional information [e.g., what aspects (domain items) should be measured] can be added if this is required for the purpose, applicability, or setting. For contextual factors, a separate list of items will be composed. Within this list, possible groups for contextual domains will be included (a) demographic variables, (b) treatment variables, (c) vitiligo history variables, and (d) pathogenetic variables.

The formulation of missing definitions of specific domain items to include in the provisional lists will rely on recommendations derived from patients/patient focus group meeting(s) and subsequent evaluation by the advisory panels. Definitions will be considered if they received an agreement score (e.g., ≥70% “agree-much agree” on a 5 or 7-point scale). In the event of multiple options, the definition with the highest agreed score will be chosen.

To support this preparative exercise, the steering committee will compile a comprehensive list that can be consulted as a help including all potential domains/domain items in order to determine what items are probably missing. The composition of this comprehensive list will be based on (1) the previously (2015) defined list of core domains and their possible domain items for trials, (2) additional literature search (including a search in ClinicalTrials.gov and review of vitiligo guidelines (from various countries if relevant)), (3) variables gathered by vitiligo experts in existing clinics and existing clinical registries, and (4) expert and patient opinions [1, 4, 12, 15, 16]. The same search strategy will be used for the list of contextual factors including the baseline characteristics and treatment-related variables. For patient-reported outcomes (PROs) included in this comprehensive list of outcomes, existing generic conceptual models of PROs and quality of life [for example the Patient-Reported Outcomes Measurement Information System (PROMIS) conceptual framework and International Consortium for Health Outcomes Measurement (ICHOM) Overall Adult Health set] and domains included in PROMs for vitiligo patients, will be used for input (CT) [17, 18]. The PROMIS framework consists of a set of outcome domains that was selected based on reviews of all existing general and disease-specific PROMs and contains the most relevant PROs across diseases.

To maintain a global view on the provisional list of items at the start of the e-Delphi rounds, the previously defined (2015) core domains/domain items that will be integrated into the list (whether in their modified form or not) will be labeled as being predefined or preselected if applicable (no voting required). All substantial modifications on the previously defined (2015) core domains/domain items will go again through the process of scoring in the first e-Delphi round together with all potential new domains and added domain items and contextual factors.

The provisional list of domains/domain items for registries and clinical practice for the e-Delphi will also rely on the previously defined (2015) core domains/domain items, but they will be integrated completely (without being labeled as preselected) in the e-Delphi scoring rounds.

##### Step 5: e-Delphi rounds - construction core dataset

The provisional lists (list for outcome domains/domain items and list for contextual factors) will be integrated in the survey (if indicated 2 separate surveys depending on feasibility aspects at that time) and will be pilot tested among members of the advisory panels before starting the e-Delphi rounds. The survey will include a maximum of 3 ranking/voting rounds. Each round will be introduced by an explanation of the study, including clear timelines and the importance of completing all e-Delphi rounds. Key terms within the survey will be explained by for instance hovering over the text. For each round reminder emails will be sent to increase the response rate.

Participants will be asked to rank each domain or contextual factor separately. They will be asked to rate the importance of the items numerically on a scale from 1 to 9 (1–3: not important, 4–6: important but not essential, 7–9: essential). This ranking procedure [according to Grading of Recommendations Assessment, Development and Evaluations (GRADE) scale (GRADE guidelines 2), and COMET handbook] may be useful to focus more on the items that are considered to be essential. It can also be helpful to elucidate or solve disagreements [19]. An “unable to score” option will be included in case the participant considers an item as unable to rate. Feedback including remaining issues can be reported by the participant at the end of each survey round.

Variables will be selected by consensus (“consensus in”) if 70% or more of 2 stakeholder groups [patient representatives (group1) versus clinicians/researchers/other stakeholders (group 2)] agree the item is essential and no strong arguments are provided against inclusion during an additional consensus meeting (= rationale against the overall trend voted as valid to overrule the 70% consensus threshold; agreement if <30% of the consensus meeting participants disagrees). Variables will be excluded by consensus (“consensus out”) if 70% or more of each stakeholder group will agree the item is not important and no strong arguments are provided against exclusion (definition of strong argument: see above). All remaining items will be categorized as “no consensus, new voting required” and will be included in the next round. Only participants who completed the previous round will be invited for the next round. We expect that 75% of the invited participants will complete the first survey and of these, 70% in the second round. Therefore, we will initially invite ±100 participants: patients (group 1: ±30 invitations) and health care professionals/researchers and other stakeholders (group 2: ±70 invitations).

##### e-Delphi rounds (step 5)

*e-Delphi rounds 1*

The first e-Delphi round will include (1) basic information of participants such as demographic data (e.g., age category, gender, country, type of stakeholder group), experience with vitiligo (for health care professionals), (2) the domain/domain items (labeled as no need to revote) from the previously (2015) established consensus process and/or obvious item*s* (such as age, gender) only to be reviewed (not to score), (3) list of domains and domain items to be scored, and (4) possibility to suggest additional domains or domain items not yet included.

*e-Delphi round 2*

In the second round, all domains/domain items will be scored again. Additional items suggested by the participants within the first round will be defined and reviewed in advance by the advisory panels/steering committee and will also be included in the second round. The second round will also include an overview of the number of participants and the distribution/percentages of scores for each domain/domain item for their particular stakeholder group and a recall of their individual scores provided in the first round. It will be requested to take the responses from other members of their stakeholder group into account before scoring again.

*e-Delphi round 3*

The third round will be introduced by an overview of the domains/domain items for which a consensus has been reached. In addition, they will receive again feedback on the scores of round 2. Distribution of the scores for each domain/domain item for all stakeholder groups will be provided as well as a recall of their individual scores. Subsequently, they will be requested to re-evaluate the scoring for all the remaining items with no consensus.

##### e-Delphi repeated for different datasets (step 6)

The e-Delphi rounds will be repeated and completed for each of the different datasets (clinical trials, registries, and clinical practice).

##### Conclusive consensus meeting (step 7)

All participants of the last e-Delphi round will be invited to attend the consensus meeting that will be organized within several months after the finalization of the last round. Participation of representatives of each stakeholder group will be encouraged. Involvement in the whole group and small group discussions will be requested to refine the discussions and voting. This meeting will be supported and guided by a non-voting methodologist or neutral observer with experience in consensus studies, to ensure that all voices are heard, to avoid the dominance of individual participants and to provide an independent oversight. The meeting will start by providing feedback on the results of the survey. Results will be presented on each e-Delphi round including response rates, information on domain/domain items with no consensus, and domain/domain items that reached “consensus in” and “out.” The remaining issues (significant disagreements between stakeholders and items with no consensus) resulting from the previous online survey procedure will be discussed. For the domains and domain items remaining inconclusive (“no consensus in or out”), additional voting will be organized by a live anonymized voting system (e.g., poll system) to analyze the results in real-time. “Consensus in” will be predefined as “if <30% disagrees.” Previous decisions resulting from the e-Delphi rounds as well as discussion to add new domains/domain items can only be reconsidered during the meeting in case of very strong/significant/convincing reasons.

If the number of domain items is considered to be too long or not feasible the consensus meeting may include an additional live voting to reduce the total number of domains/domain items.

#### Part 2

Part 2 will focus on “how to measure (instrument),” “when to measure,” “what scale to use,” and instructions to report the data. This part will be based on recommendations of the HOME initiative, Outcome Measures in Rheumatology (OMERACT) filter, and COSMIN guidelines.

##### Step 8: Quality assessment of instruments

This step will include the quality assessment of the instruments. Information provided within this step will be important for the subsequent selection of the instruments in step 9. The extent and quality of the measurement properties (validity, reliability, responsiveness) of existing physician-reported outcome measures, PROMs, and imaging techniques for vitiligo will be evaluated according to the COSMIN methodology (www.cosmin.nl). Results will be collected in a systematic review-based method. The studies evaluating the measurement properties of instruments will be selected and the quality of the instruments will be critically evaluated.

For the timing (when to measure) and “type of scale and instructions for reporting the data,” information provided by the literature (including the original report of the instrument and the possible modified use in subsequent vitiligo studies) and expert experience will be collected.

##### Step 9: Selection of measurement instrument, scales, timing, and instructions for reporting

A draft proposal for instrument selection per domain/domain item per setting, timing, and scale will be created by the advisory panels by consensus ( ≥70% agreement) based on evidence from the validation studies, information on the quality of the instruments provided in step 8 as well as other relevant literature (ref 6), expert experience/opinions, feasibility aspects, use in existing databases (e.g., local/national registers), the measurement instrument's potential for use in different countries, the number of available translations, availability of the instrument, time requirements, and practicability. To the greatest extent possible, only instruments that had been validated will be considered. Instructions for “how to report the outcomes” will be developed using the guidelines of the EQUATOR network.

The evidence for each proposed measurement instrument will be presented via PowerPoint presentation and electronic/paper documents during a consensus meeting that is open for all participants of part 1 as well as additional stakeholders. Subsequently, this proposal will be discussed by the whole group and small group discussions during the meeting(s) (live and/or virtual). These consensus meeting(s) will also include voting (live anonymous voting system or electronic survey) to obtain a consensus. A predefined consensus rule of ≥70% agreement will be used for selecting core measurement instruments and related discussions will be iterative until full agreement is reached. More than one instrument may be selected for a specific domain item if a legitimate choice exists to choose different options (e.g., 2 comparably validated instruments displaying different strengths and weaknesses depending on the measurement property). All decisions will be based on feasibility and current best practices.

To determine when domain items should be measured (“when to measure”), an online survey will be used. The voting options will be derived from current clinical practice and clinical trials (e.g., ClinicalTrial.gov). Consensus on the “when to measure” will be based on the majority of the votes for one of the options.

Specific gaps within this field will be identified. If required, additional validation studies may be initiated and in case a specific tool is still missing, the development of a new instrument may be considered. Certain details for these and other additional steps will be worked out later in an additional protocol if necessary.

##### Step 10: Final conclusive meeting

During a final conclusive meeting, an overview of the 3 completed core datasets (core outcome sets and contextual factors) for the 3 different settings including domains/domain items (part 1) and instruments (part 2) will be presented to the participants of all 3 settings*.* In addition, the future dissemination plan and implementation goals will be defined.

## Discussion

One of the most important goals of evidence-based medicine is to achieve an international consensus on a single core set of data to be collected in clinical practice, trials, and registries. The final result of the agreed core datasets will enable all researchers and clinicians to collect data in a standardized and harmonized way. This will stimulate collaboration, facilitate communication between research groups, and enhance the comparability between different studies and clinical trials. The use of these uniform core data will ultimately lead to a better understanding of the pathogenesis of vitiligo, treatment response, and long-term results.

By following a structured approach starting from a provisional list which includes data from the available literature, the opinions of vitiligo experts and patients, and by using the previous (2015) defined core domains as a foundation, we plan to select relevant domains and domain items by e-Delphi technique. Subsequent consensus meetings will be used for the selection of outcome instruments.

After this is completed, the core datasets (core outcome sets and contextual factors) will need to be tested for feasibility in different countries and cultures and existing database infrastructures. To facilitate this procedure, a template (electronic and paper versions) for uniform reporting of data in vitiligo trials, clinical practice, and registries will be created and provided to clinicians and researchers. Moreover, this will be supplemented by instructions for use and other training materials. If issues arise, modifications may be implemented. The consensus-based core dataset(s) can subsequently be integrated into national and international collaborative efforts, including existing prospective databases and registries, and be available for use in trials and daily clinical practice. Future reviews of the datasets will be important on a periodic basis to ensure the items included are still relevant, to include possible new outcomes or outcome measures, to evaluate the status of implementation, and to engage additional stakeholders.

### Study status

The project is currently in steps 1–3 (part 1). The protocol has been approved, scope and applicability are defined, and the participants will be selected.

Protocol versions: Version 1: 26/02/2020; version 2: 01/04/2020; version 3: 14/05/2020; version 4: 29/06/2020; Version 5: 02/07/2020; Version 6: 20/08/2020; Version 7: 25/08/2020; version 8: 31/08/2020; version 9: 18/01/2021); version 10: 15/03/2021; version 11: 18/04/2021; version 12: 07/09/2021; version 13: 17/10/2021; version 14: 17/11/2021; version 15: 30/12/2021; version 16: 26/01/2022; version 17: 08/02/2022; version 18: 29/05/2022

Start date of the project: initiation October 2019

## Data Availability

Not applicable

## Abbreviations

C3: Consortium for Harmonizing Outcomes Research in Dermatology (CHORD) - Cochrane Skin - Core Outcome Set Initiative (CS-COUSIN) - collaboration
CHORD: Consortium for Harmonizing Outcomes Research in Dermatology
COI: Conflict of interest
COS: Core outcome sets
COMET: Core Outcome Measures in Effectiveness Trials
COPE: Committee on Publication Ethics
COSMIN: COnsensus-based Standards for the selection of health Measurement Instruments
CS-COUSIN: Cochrane Skin - Core Outcome Set Initiative
EMA: European Medicines Agency
FDA: Food and Drug Administration
HOME: Harmonising Outcome Measures for Eczema
ICHOM: International Consortium for Health Outcomes Measurement
INFO: International Initiative for outcomes for vitiligo trials
NGT: Nominal group technique
PROMIS: Patient Reported Outcomes Measurement Information System
PROMs: Patient-reported outcome measures
PROs: Patient-reported outcomes
VIS: Vitiligo International Symposium
VETF: Vitiligo European Task Force
VGICC: Vitiligo Global Issues Consensus Conferences
VIPOC: Vitiligo International Patient Organizations Conference
VITAL: Vitiligo International Task Force for an Agreed List of core data
